# Listening to voices from multiple sources: A qualitative text analysis of the emotional experiences of women living with breast cancer in China

**DOI:** 10.3389/fpubh.2023.1114139

**Published:** 2023-02-03

**Authors:** Chaixiu Li, Cathy Ure, Wanting Zheng, Chunrao Zheng, Jianhong Liu, Chunlan Zhou, Biao Jian, Lijun Sun, Wenji Li, Lijun Xie, Yuchang Mai, Huihui Zhao, Yusheng Liu, Jie Lai, Jiaqi Fu, Yanni Wu

**Affiliations:** ^1^Department of Nursing, Nanfang Hospital, Southern Medical University, Guangzhou, Guangdong, China; ^2^School of Nursing, Southern Medical University, Guangzhou, Guangdong, China; ^3^Directorate of Allied and Public Health, School of Health and Society, University of Salford, Salford, Manchester, United Kingdom; ^4^Vascular Thyroid Breast Surgery Center, Affiliated Hospital of Guangdong Medical University, Zhanjiang, Guangdong, China; ^5^Department of General Surgery, Shenzhen People's Hospital, Shenzhen, Guangdong, China; ^6^Office of Retirement Work, The Second Xiangya Hospital of Central South University, Changsha, Hunan, China; ^7^Software Quality Engineering Center, China Electronic Product Reliability and Environmental Testing Research Institute, Guangzhou, Guangdong, China; ^8^Reliability and Environmental Test Engineering Center, China Electronic Product Reliability and Environmental Testing Research Institute, Guangzhou, Guangdong, China; ^9^Department of Breast Surgery, Nanfang Hospital, Southern Medical University, Guangzhou, Guangdong, China

**Keywords:** breast cancer, emotional experience, Chinese women, text analysis, qualitative study, psychological support

## Abstract

**Background:**

Receiving a breast cancer diagnosis and treatment is both a physical and emotional journey. Previous studies using single-source data have revealed common and culture-specific emotional experiences of patients living with breast cancer. However, few studies have combined such data from multiple sources. Thus, using a variety of data sources, the current study sought to explore the emotional experiences of women in China newly diagnosed, post-operative, or undergoing chemotherapy. We posited that even though women living with breast cancer in China have multiple channels through which they can express these emotional experiences, little variance would be found in their emotional expressivity and the themes they want to express due to cultural inhibitions.

**Methods:**

Text data from female patients newly diagnosed, post-operative, or undergoing chemotherapy were collected between June 2021 and January 2022 *via* a Python web crawler, semi-structured interviews, and an expressive writing intervention. Data were transcribed and subjected to thematic analysis. Reporting followed the consolidated criteria for reporting qualitative studies (COREQ) guidelines.

**Results:**

Analyses were based on 5,675 Weibo posts and comments published by 448 posters and 1,842 commenters, transcription texts from 17 semi-structured interviews, and 150 expressive writing texts. From this total collection of 461,348 Chinese characters, three major themes emerged: (i) conflicting emotions after diagnosis; (ii) long-term suffering and treatment concerns; and (iii) benefit finding and cognitive reappraisal.

**Conclusions:**

Despite gathering information from various sources, we found that distress from body-image disturbances, gender role loss and conflict, and changes in sexuality and fertility, were consistent among this sample of female Chinese patients with breast cancer. However, when women engaged actively in benefit finding and cognitive reappraisal with strong social support, patients were able to find ways to adapt and reported post-traumatic growth. Strong social support was an important facilitator in this growth. These study findings emphasize that healthcare professionals ought to increase cultural sensitivity, provide multiple channels to encourage patients to express their emotions, and incorporate screening for patients' emotional distress at all diagnostic and treatment phases as part of routine nursing care.

## 1. Introduction

Breast cancer (BC) is the most common cancer in women worldwide with an estimated 2.26 million new cancer cases diagnosed in 2020 (1). Accounting for 15.5% of female cancer deaths worldwide, it has also become a heavy global public health burden (1). BC diagnosis and treatment are stressful experiences that can evoke a variety of negative emotions and lead to broader emotional experiences (2, 3). “Emotional experience” encompasses an individual's emotional reactions and associated cognitions regarding relevant events, including thoughts, feelings, worries, and concerns (4).

A BC diagnosis usually occurs when women are at a life stage often characterized by childrearing and being mid-career, and when they may be less able to cope with the risk of lifelong cancer recurrence or potential death (5). Treatment side effects and prognostic uncertainties cause women to suffer negative emotional experiences, including stress to their significant relationships (6, 7), body-image disturbance (8, 9), sexuality and fertility anxieties (10), social stigma (11), confusion about recurrence and hereditability (12), and negative quality-of-life (5). Despite this, previous studies have also reported some positive emotional experiences, including post-traumatic growth (13, 14) and self-compassion (15).

BC diagnosis and treatment is both a physical and emotional journey. One study exploring individuals' psychosocial wellbeing across disease phases indicated that distress peaks occur after diagnosis, during chemotherapy, after mastectomy, at the end of adjuvant therapy, at diagnosis of recurrence, and when the disease is declared to be terminal, highlighting that patients across different BC diagnostic and treatment phases can experience a marked range of emotions and cognitions (5, 12, 16–19). However, most research has focused exclusively on patients who are newly diagnosed, those receiving chemotherapy, or across a variety of durations post-diagnosis (5, 8, 10, 11), which may neglect treatment continuity and integrity.

Although previous studies provide valuable insights into Chinese women's culture-related views on BC, most have focused on Chinese immigrants in other countries (20–23). Those have found that, as Chinese immigrants acculturate to the mainstream culture of other countries, they may incorporate new post-immigration cognitions (20, 21). Specifically, those in Western cultures tend to value free open emotional expression; in contrast, those in Eastern cultures tend to value emotional suppression, or an individual's ability to consciously control their emotional expressions (20–23). From a Chinese cultural perspective, free expression of emotions, especially negative emotions, may temporarily disrupt group harmony (21, 23). Chinese women especially are conflicted about disclosing emotional distress and experience high levels of ambivalence about doing so (21, 23). In Chinese culture, cancer is often perceived as misfortune and contagious, which has been associated with self-stigmatization (24). Fear of cancer being discovered and bringing shame or burden to the family leads patients to prioritize emotional suppression to preserve social harmony (22). Meanwhile, socially constrained responses may be negatively associated with relationship satisfaction, aggravate self-stigmatization, and result in persistent emotional distress and reduced self-efficacy in coping with stress (3, 21). Consequently, this context is not conducive to healthcare professionals and caregivers' timely identification of patients' emotions or their ability to provide corresponding emotional support.

Numerous published studies have shown that expressive writing (EW) and interviewing are effective ways to record patients' experiences through hearing their voices (2, 7, 25). Furthermore, social media has been recognized as another potential source of important patient data. Platforms such as Facebook, Twitter, and Weibo, are all based on the sharing, dissemination, and acquisition of user relationship information, where short, real-time information can be obtained through attention mechanisms (26). In the Internet age, social media has emerged as a rich and largely untapped resource for understanding patients' frank communications about their experiences and treatments (26–28). Despite the advantages of these data, their limitations also warrant mention as they may introduce bias in identified experiences. First, face-to-face interviews may skew the sample toward participants who find it easy to talk about their experiences with strangers, while those in serious emotional distress may refuse to participate (29, 30). Second, although using EW as a method to collect the experiences of patients with BC is effective, such patients must be physically able to write by hand for more than 20 min; those with physical disorders like lymphedema or those whose surgery occurred on the same side as the hand used to write are thus ineligible (31). Our previous research has also identified that not all patients with BC like expressing their emotions in writing; there are individual differences in how feelings are expressed in different ways (e.g., verbally or through facial and body cues) (25). While emotional expressions are made on social media in the real world and in real-time, the patients' demographic and disease characteristics are incomplete or unavailable on these platforms (27, 28). Arguably, therefore, it is difficult to capture the full range of nuances in patients' disclosures of their emotional experiences from a single data source, especially concerning women living with BC in China, where cultural norms strongly discourage emotional disclosure (27, 32). Use of a single-source method would likely skew results and fail to reflect the target population's comprehensive range of experiences. To date, few studies have explored patients' experiences using multiple sources.

Supporting and accompanying female patients with BC throughout their cancer journey requires a deep understanding of their emotional experiences. Therefore, we conducted this qualitative text analysis based on data collected from patients during three BC phases: when they were newly diagnosed, postoperatively, and while undergoing chemotherapy. The study goal was to help healthcare professionals provide individualized culturally appropriate emotional support and management for female Chinese patients living with BC.

## 2. Methods

### 2.1. Study design

The aim of this study was to explore the emotional experiences of women newly diagnosed, post-operative, or while undergoing chemotherapy for BC in China. Qualitative data was collected from Weibo, semi-structured interviews, and an expressive writing intervention. This qualitative study uses the consolidated criteria for reporting qualitative studies (COREQ) (33), and was analyzed based on the data collected from Weibo, EW, and semi-structured interviews (Supplementary material 1).

### 2.2. Participants and settings

EW participants were recruited from the breast surgery departments of six tertiary hospitals in four cities in China. Semi-structured interview participants were selected from one of the above hospitals and they did not participate in EW. Weibo participants were selected from among network users within the column of the super topic *#Breast cancer#*.

The inclusion criteria for patients with BC included in the EW and semi-structured interviews are presented in Table 1. Consistent with the requirement to include maximal variation during purposive sampling for qualitative text analysis, we recruited 50 EW participants undergoing each of three BC phases: newly diagnosed, postoperative, or undergoing chemotherapy (34–36). Furthermore, with respect to the potential differences in phase specificity and acuteness of patient emotions, newly diagnosed and postoperative were defined as within 1 month of a new diagnosis and 1 month post-surgery, respectively, consistent with previous studies (19, 37). Since participants from Weibo are anonymous and we could thus only analyze the texts they disclosed on the Internet, we were unable to apply inclusion criteria to this sample. The EW and semi-structured interview participants' demographic characteristics are shown in Table 2.

**Table 1:** Inclusion and exclusion criteria for patients included in the expressive writing and semi-structured interviews.

**Inclusion criteria**	**Exclusion criteria**
• Female patients diagnosed with breast cancer by pathology	• With any type of psychosis, or mental or auditory deficit
• Age over 18 years	• Having other tumors or other major diseases
• Patients were conscious and could communicate frequently	
• Patients were able to write Chinese and physically able to write by hand for more than 20 min at a time (For expressive writing patients)	
• Participants within 1 month of diagnosis, 1 month after surgery, or undergoing chemotherapy	
• Voluntary participation and agreed to sign the informed consent form	

**Table 2:** The demographic characteristics of participants in expressive writing and interviews.

**Characteristics**	***N* (%)**
**Age (years)**	
18–29	8 (4.8)
30–39	54 (32.3)
40–49	69 (41.3)
50–59	32 (19.2)
≥60	4 (2.4)
**Cancer stage**	
I	45 (26.9)
II	90 (53.9)
III	27 (16.2)
IV	5 (3.0)
**Educational level**	
Primary school or below	22 (13.2)
Junior high school	63 (37.7)
High school	52 (31.1)
University or above	30 (18.0)
**Occupation**	
In-service	83 (49.7)
Retired	18 (10.8)
Unemployed	49 (29.3)
Farming	17 (10.2)
**Marital status**	
Married	156 (93.4)
Single	6 (3.6)
Divorced	5 (3.0)
**Faith**	
No	145 (86.8)
Buddhism	16 (9.6)
Christian	2 (1.2)
Other	4 (2.4)
**Types of medical expense payment**	
Free medical treatment	4 (2.4)
Medical insurance	151 (90.4)
Self-pay	11 (6.6)
Other	1 (0.6)
**Economic burden**	
Heavy	76 (45.5)
Medium	81 (48.5)
Low	10 (6.0)

### 2.3. Data collection

EW and semi-structured interview participants were informed about the standard EW instructions and interview guide, respectively (see details in Table 3). Patients were approached in their hospital rooms by four trained female nurse researchers (referred to herein as: CL, WZ, CZ, and JL) who are experienced in qualitative methods and were part of the BC department staff of their respective hospitals. EW participants were asked to write about their most prominent stress-related, upsetting, or traumatic feelings caused by BC for at least 20 min over four consecutive days at their convenience in accordance with the standard EW by Pennebaker and Beall (38). If their EW materials were not received within 1 week, the research assistant at each hospital asked the participants whether they had encountered difficulties in the writing process and whether they were able to complete the writing task. The 50 EW texts from participants in the chemotherapy phase were randomly selected from our previous study, a multicenter randomized controlled trial on the effect of prolonged EW on patients receiving chemotherapy (31). The semi-structured, face-to-face interviews were conducted by an experienced female researcher (CX L) at the patients' convenience in a quiet conference room. Each interview lasted 30–40 min and the audio was digitally recorded. Semi-structured interview participant recruitment ended when data saturation was achieved, which was when no new information emerged (39).

**Table 3:** Pennebaker's expressive writing instructions and interview guide.

**Pennebaker's expressive writing instructions**	**Interview guide**
Advise your patients to: • Find a place and time where and when you will not be disturbed. • Write about what you are worrying about or what you have been avoiding thinking about that is affecting your life in an unhealthy way. • Write for a minimum of 20 min a day for at least 4 consecutive days. • Write continuously without worrying about proper Chinese or censoring content. • Decide whether to write about the same issue each time or different issues. • Consider reviewing what you are written over time to see how your thinking or emotions have changed. • The information obtained will only be used for this research and will not have any influence on you.	• “Please tell me about your feelings during this stage of your diagnosis. Did you have any thoughts or confusions?” (For newly diagnosed phase patients) • “Please tell me about your feelings before and after the operation. What adverse symptoms or experiences did the operation result in for you?” (For post-operative phase patients) • “Please tell me about your feelings when you began to receive or during the chemotherapy. What adverse symptoms or experiences did chemotherapy result in for you?” (For chemotherapy phase patients) • “What was your feeling during the whole period of illness or treatment?” (For all patients) • “Do you have any new experiences, feelings, or views recently? What things or symptoms have changed your mood?” (For all patients)

The data generation and collection were synchronized. With the exception of the EW texts by patients in the chemotherapy phase being obtained from our previous research data, the rest of the EW, interviews, and Weibo texts data were generated and collected from June 2021 to January 2022. We conducted 17 interviews and transcribed the recordings immediately following each interview. In the process of EW data collection, we identified some common invalid contents. For example, some participants did not finish the four-day continuous writing, or their written content was almost an expression of gratitude to and praise of the medical staff. Therefore, every time a document was collected, we would check its content validity so that we could recruit new participants in time if we found that the content was unqualified within the study period. Thus, EW data collection was synchronized for completion from June 2021 to January 2022. Posts and comments published by Weibo participants from June 2021 to January 2022 were obtained using a Python web crawler.

### 2.4. Data analysis

Thematic analysis, as outlined by Braun and Clarke, was used to identify themes, similarities, and differences between participant experiences (40). Analyses of these large qualitative datasets were carried out using a structured approach (41). Electronic texts from EW, interviews, and Weibo were regarded as three separate datasets. We performed double screening (by CL and JF) to manually exclude contents that were obviously not self-reported by patients with BC (e.g., posts by daughters asking questions on behalf of their mothers, or doctor's explanations of BC-related knowledge).

Each dataset was imported into NVivo (Version 11.0; QSR International) to allow for electronic coding. First, one researcher read and coded each of the three datasets. Then, a second researcher familiarized herself with a random sample from each dataset, after which the first and second researchers discussed and agreed on coding decisions. Next, three authors compared, contrasted, consolidated, and grouped these codes into categories according to their similarities, organized these categories into themes, and identified quotations that represented each theme. Discrepancies and disagreements were discussed by the team and adjusted as needed. In the final step, all authors evaluated the findings and agreed upon the most salient themes to be highlighted herein.

### 2.5. Ethical considerations

Ethical approval for this study was obtained from Nanfang Hospital of Southern Medical University in China (NFEC-2021-124), and a standardized informed-consent form was administered. Eligible EW and semi-structured interview participants were informed about the study and provided written informed consent prior to participation. During the writing and interview processes, an experienced psychologist was available for patient consultation upon request. Codes were assigned to each participant instead of their real names (i.e., PD1–50: newly diagnosed phase participants; PO1–50: postoperative phase participants; PC1–50: chemotherapy phase participants; PW1–5,675: Weibo participants; PI1–17: semi-structured interview participants).

### 2.6. Rigor

To establish study credibility, all researchers engaged long-term with and established appropriate relationships with the participants. To enhance study dependability, any disagreements on the design, methods, data analysis, or results were discussed by the research team until consensus was reached. To ensure confirmability, the researchers used field notes and memos to support the connections between the data and findings. To guarantee authenticity, all major identified themes and sub-themes were supported by participant quotes. Transferability was accomplished by including the widest participant diversity possible, and the authors sought to describe all parts of the study methods in detail to guide future studies.

## 3. Results

Materials from 17 semi-structured interviews and 150 EW texts from female patients with BC were collected from across the three BC phases. After removing the contents unrelated to the self-report of BC patients, a total of 5,675 Weibo posts and comments published by 448 posters and 1,842 commenters were obtained. The texts from these three sources represented a total of 461,348 Chinese characters. During the EW and interview processes, no participants sought counseling or psychotherapy. Three major themes comprising eight sub-themes were identified from the texts: (1) conflicting emotions after diagnosis; (2) long-term suffering and treatment concerns; and (3) benefit finding and cognitive reappraisal. Themes, sub-themes, and representative participant quotes are shown in Supplementary material 2, along with the number and proportion of participants who mentioned each theme and sub-theme. The proportion of themes and sub-themes mentioned in each source is shown in Supplementary material 3.

### 3.1. Conflicting emotions after diagnosis

Receiving a BC diagnosis changed patients' views of the future, as it required that they consider the potential for losses, unrealized goals, and death, while they simultaneously considered the feelings of their loved ones; this immediately increased the risk of stress-related disorders for patients. The participants also shared experiences of diagnosis-specific distresses, such as concerns about diagnosis disclosure (*n* = 158, 6.4%), disbelief and escapism (*n* = 187, 7.6%), and treatment decision-making distress (*n* = 459, 18.7%); they were also concerned that these experiences would persist and have implications for their mental health and adherence to follow-up treatment.

#### 3.1.1. Concerns about disclosing cancer diagnosis

Disclosing one's inner pain to a trusted other may help relieve stress and ease anxiety. While bewildered by their inner conflicts over disclosure, several newly diagnosed participants tended to withhold their cancer-related distress for fear of burdening others, especially their older parents. One participant said that:

*I dare not disclose my cancer diagnosis to my parents. I'm worried that they can't accept this shocking news, and that their bodies can't bear this pain*. [PD18]

In addition, some participants reported an unwillingness to disclose because they did not want to receive excessive attention or suffer being stared at or gossiped about by others. As one stated:

*If everyone knows the news, their various concerns and sympathies will be overwhelming to me, and I am afraid that I will become more vulnerable instead*. [PW31]

#### 3.1.2. Disbelief and an escape from reality

Disbelief of the diagnosis was particularly common among participants who had annual physical examinations and who felt well prior to their diagnosis, as they could not reconcile a cancer diagnosis with their healthy identity. Strong disbeliefs even led some participants to attribute their cancer to having done something bad:

*What? Cancer? I was scared because I never thought cancer would have anything to do with me. I was a timid and kind-hearted person and I had never killed a chicken or a cockroach. I couldn't believe it!* [PD2]

Moreover, some participants deliberately turned their attention to things other than the disease, such as traveling, shopping, and other interests to escape the reality of being a cancer patient who may die. One participant said:

*I have been pretending to be strong since diagnosis. I want to paralyze myself [sic] by going shopping, going to the movies … but in vain, my heart is always full of fear, anxiety, sadness, and hopelessness..*. [PC4]

Some participants mentioned that when some warning signs of BC appeared in their bodies, they did not pay attention to them, and because of their blind belief that they did not have cancer, they delayed medical treatment for careful examination and regular check-up.

*There was a lump in my left breast, and I did not feel anything abnormal, so I did not believe it would turn into BC. However, when I went to the hospital for reexamination after a long time, the results made me feel hopeless*. [PI2]

#### 3.1.3. Distress over rapid treatment decision-making

Surgery type, especially mastectomy, is considered a “preference decision” for newly diagnosed patients who are clinically eligible for either option (42). These patients must weigh risks and benefits of treatment options on multiple grounds. Concerns regarding cancer recurrence, prognosis, personal costs, benefits, and other salient aspects of surgery play crucial roles in the decision-making process, which can cause intense distress.

Mastectomy posed a greater threat to participants who were young and unmarried or married but childless and did not want to be deprived of their rights as a partner, wife, or even mother. They were afraid of being a “monster” in others' eyes. One single participant complained that:

*My sister has been persuading me to receive mastectomy, but I have no boyfriend, no husband, and no children. What will happen to me in the future? Certainly, no man wants to spend his whole life with an incomplete woman*. [PO45]

Most participants who had originally preferred breast-conserving surgery eventually chose the mastectomy based on fear of prognosis, recurrence, financial burden, long follow-up treatment, and a strong will to live, with the accompanying belief that “*nothing matters except my life*”. One participant said:

*I'm afraid of recurrence after breast-conserving surgery. Life is more important for me, so I will directly choose [sic] the mastectomy. If my husband minds my incomplete breast and abandons me, I will also not care. I just want to live for myself*. [PI7]

Interestingly, we found that some participants felt confused about where to receive treatment and from whom to receive it. Some tended to look for the hospital with the greatest expertise in more developed cities to ensure a cure. One participant who traveled from afar stated:

*I just planned to have an operation in my hometown, but some friends and relatives advised me to go for the excellent medical skill and condition [sic]*. [PI13]

### 3.2. Long-term suffering and treatment concerns

Diagnosis is often followed by surgery or chemotherapy. The realization that they must begin treatment and/or the impacts of treatment was extraordinarily stressful for most participants. The special treatment-related concerns that caused suffering for female patients living with BC throughout treatment included body-image disturbances and sense of stigma (*n* = 277, 11.3%), anxiety over sexuality and fertility changes (*n* = 280, 11.4%), and guilt and powerlessness over loss and conflict in their gender roles (*n* = 396, 16.1%).

#### 3.2.1. Body image disturbance and sense of stigma

Body-image changes like breast loss or disfigurement, hair loss, scars, and increased weight undoubtedly resulted in a sense of body-image disturbances, which can have devastating effects on women's identity, attractiveness, self-esteem, sexuality, and social adjustment.

Hair loss can be a traumatic experience for some women as it is suggestive of cancer, which can be stigmatizing, and may result in feelings like embarrassment, sadness, shame, helplessness, and a sense of impending death. Several participants declared that they preferred to cut off all their hair to avoid the dreadful process of hair loss:

*I keep losing hair! Is my physical condition getting worse and worse, and am I going to die? It's horrible! So it's better to shave it off to avoid it falling all over the floor*. [PC17]

Breast loss, scarring, and deformity associated with surgery may lead participants to feel that they are no longer normal women, but instead embarrassed, ugly, and self-conscious. They were afraid of looking at themselves in mirrors and of being looked at as peculiar and being discarded by their intimate partner. One participant who had a mastectomy said:

*I was desperate when I saw my surgical scar in the shower. I felt I was no longer a perfect woman, but a defective product. I didn't want my husband to see my ugly appearance, I hate myself*. [PO42]

#### 3.2.2. Guilt and powerlessness over gender role loss and conflict

Gender roles changing from participants' original identities (e.g., mother, wife, daughter, employee) to patient was a great pressure. Participants mentioned a sense of guilt over losing their original role, the crisis of self-identity, and low self-efficacy over the conflict between their original role and new patient role.

Participants with older parents and younger children experienced more obvious distress, because they had changed from the main family caregiver to the cared for, a change that made them feel deeply guilty for making their families suffer and led them to label themselves as “unfilial”, “useless”, and “a burden”. One participant said:

*My children are still young, and my parents are old. I'm so unfilial, I failed to give them a good life, and even have this disease, which will only make them worry*. [PO23]

Breastfeeding and caring for a newborn are conducive to establishing a close mother-child relationship. Yet accomplishing this can be impossible for these women, some of whom were in the lactation period. A participant who had just given birth said:

*My child is only 3 months old, but I can't breastfeed him, accompany him out, and take care of him like other children's mothers. I can't help crying when I miss my child*. [PD45]

Career impacts caused by BC, such as delayed work schedules, job changes, and more seriously, unemployment, also bothered participants. Being away from work for long periods made them feel that life was aimless and hopeless. One participant expressed:

*Now I don't have to go to work because of illness, I'm just like a gyroscope losing its inertia or a mobile phone without electricity. It is extremely miserable for me, who relies on work to gain a great existence [sic]!* [PC17]

Notably, regardless of whether the real cause was discrimination, dismissal of an employee suffering from BC undoubtedly caused self-identity crisis. A participant who was dismissed from their company for no apparent reason recalled that:

*I was dismissed, which was too hurtful. I asked my boss the reason, they bluntly said it was just because the company laid off employees, and I was ill, so they fired me…* [PW1170]

That patients are physically and psychologically vulnerable during treatment, and that they are unable to take on the same responsibilities as mothers, wives, and daughters as before deserves special attention. If they perceive inadequate family support or cannot be understood patiently, they form a sense of self-perceived burden and even consider suicide. One participant said:

*When I quarreled with my husband, he scolded me, “Why did you get this disease? Didn't you know that you couldn't get sick?” He is right! I have no right to get sick! I should die!* [PC19]

#### 3.2.3. Anxiety about sexuality and fertility changes

Women with BC experience a range of negative emotional changes because of disturbances to their sexuality, including fear of relationship deterioration, loss of fertility, sexual unattractiveness, loss of femininity, depression, and anxiety, as well as alterations to their sexual self. A few participants mentioned that, since beginning treatment, both their sexual desire and frequency of sexual intimacy had obviously decreased; some even felt indifferent to their sexual life:

*Since the operation and chemotherapy, I have been indifferent to sex. I usually have sex two or three times a month, but now once is more than enough*. [PC20]

Despite a low sexual desire, some participants still wanted information related to having sex. Yet there were also indications that they forewent counseling for fear of shame. One participant stated:

*I don't know if I can have sex after surgery. I feel that my sexual desire is gone. Is it really that I can't have sex for several years? I really want to ask the doctor, but it's too embarrassing to mention it*. [PO36]

Sexual dysfunction from treatment is a difficult hurdle for participants who want to get married or have children. They are also confused about the answers to questions like “Can I get pregnant after treatment?” and “When can I get pregnant?”. Yet, interestingly, the detailed aspects of fertility anxiety varied among participants with different marital statuses. Those who were unmarried and childless became utterly dispirited and depressed over future relationships and marriage. A very young participant complained that:

*I don't have a boyfriend and I'm not married. How can I accept the fact that I am a young girl who has no breasts? What should others think of me? Who would choose a crippled woman? I'm a waste... I'm going crazy because of my body disfigurement*. [PO25]

Those who were married but childless had worries concerning fertility and guilt about their husbands, as they thought they had not fulfilled their responsibilities as a wife and feared being abandoned. Similarly, influenced by the traditional Chinese construct of “son preference”, these negative emotions were more serious among participants who were married with only a daughter. Despite going to painstaking efforts to conceive, BC diagnosis during pregnancy means that patients may also face a serious choice between the fetus and themself. A participant reported that:

*The only dream that will come true soon is giving birth to a boy. But now I have to terminate my three-month pregnancy. It is simply unacceptable! I think I may not be able to give birth to him and I may die soon. So suffocated [sic]!* [PC29]

### 3.3. Benefit finding and cognitive reappraisal

In addition to painful memories during diagnosis and treatment, participants also recorded expressions of benefit finding and cognitive reappraisal regarding their BC. They realized the importance of their health and planned to reestablish their life goals to really live for themselves. They also noticed that there were many around them who wholeheartedly accompanied them, which made them feel grateful and inspired to put effort into surviving their painful journey.

#### 3.3.1. Post-traumatic growth

Once they had overcome the initial shock of diagnosis and painful treatment-related side effects, participants realized the importance of treatment to survival and the process of healing was initiated. Many (*n* = 582, 23.7%) expressed that they had also experienced positive psychological growth following this traumatic event, and tended to change their negative mindset, cooperate with treatment, and strive for their “new birth”, instead of being afraid and wanting to escape.

A few participants regarded the cancer as a rest and welcomed being given a chance to reflect on their priorities and focus on themselves. Some also stated the importance of a correct understanding of their new roles and offered suggestions that reflected both their growth through the illness process and a newly significant way of conveying positive emotions. One participant suggested:

*Don't think of yourself as a patient. You can cook some delicious food for your family, clean up your home, grow some flowers … treat yourself as being on a holiday and spend more time with your family*. [PW35]

Some also began to establish more positive cognitions about diseases and attach greater importance to the subtle changes in their bodies. One participant said:

*Regret is useless. I would like to appeal to those who seem to be very healthy. Once you find anything wrong with your body, you should go to the hospital for examination immediately. Do not hesitate*. [PC19]

#### 3.3.2. Perception of social support

Many participants (*n* = 166, 6.8%) acknowledged that suffering can be reduced with sufficiently supportive national policies, family, friends, healthcare professionals, and peer patients. Participants were extremely grateful for their company, encouragement, and sound guidance. Such support was often viewed as a behavioral manifestation of emotional support, which largely strengthened their confidence in defeating cancer. Some participants, particularly those with heavy family burdens, indicated that they were grateful for the national health insurance reimbursement policy. One said:

*It's not affordable for an ordinary worker to have the extra treatments. Fortunately, under the guarantee of national medical policy [sic], my financial burden seems to have been reduced a lot*. [PW226]

Family support was deemed as the most important source of social support. Participants' main caregivers were almost always their husbands, mothers, or children. Many realized that the tireless care and intimate company of family gave them strong confidence and motivation to persist. In their view, only by adhering to treatment and recovering early could they live up to their family's efforts. One participant said:

*I have become a fragile baby in my family since I was ill. My son will not make me angry. Besides, my husband tries every means to make me happy! I think, no matter how hard the process is, I must persist!* [PC35]

The effectiveness of peer patient support was also mentioned frequently. Participants admitted that they could better understand each other's feelings and share experiences about fighting side effects to help reduce the misery of those who were in treatment. One participant stated that:

*All the patients were full of warmth. It is easier for us to understand and sympathize with each other. We often chat together to encourage and take care of each other, as if we have become a family*. [PO15]

## 4. Discussion

Prior studies addressing the emotional experiences of patients living with BC used single-source data (7, 26, 42–44). This study adds to the literature by providing insights from textual data collected from multiple sources and across different treatment phases. Despite gathering information from multiple sources, there is little variance within the theme of self-reported emotional experience presented by these women with BC in China. We contend that cultural inhibitions play a significant role here given women in China are encouraged to suppress their emotions (3, 22, 45). The multifaceted experiences were identified as representing three consistent themes that comprise eight sub-themes: (i) conflicting emotions after diagnosis; (ii) long-term suffering and treatment concerns; and (iii) benefit finding and cognitive reappraisal.

Our findings indicate that patients suffer painful emotional conflicts regarding both disclosing their cancer diagnosis and rapid treatment decision-making. In China, cancer is predominantly considered a result of karma or bad luck caused by the immoral behavior of individuals or their ancestors (43). The value orientation of Chinese traditional culture focuses on neither spiritual sustenance nor material wealth, but on coordinating interpersonal relationships and creating a harmonious society (20, 23). Within a cultural atmosphere of “faithfulness and honesty” that advocates ethics, feminism—which emphasizes individuality—is doomed; this cultural system that stresses integrity and emphasizes balance further ignores women's personal needs (45). Interestingly, and consistent with previous studies (2, 20, 24), many participants herein shared that they prioritized emotion suppression to preserve social harmony and were tempted to keep their disease secret as they were conscious of the burden and shame that cancer would impose on their families, and were afraid of the staring, gossip, and excessive sympathy they would receive (20, 24). Within such a cultural milieu, patients feel that having BC is unimportant to others and unnecessary to share. Thus, some participants stated their unwillingness to disclose their cancer-related issues and true thoughts in public. Nevertheless, more frequent suppression of emotions and emotional expression tends to be associated with greater psychological distress and poorer interpersonal functioning (3, 23). Consistent with other studies (46, 47), we found that most participants ultimately chose the relatively simple surgical option (mastectomy) based on greater perceived survival expectations and faster hospital discharge, rather than physical appearance. Similar to Mazaheri's study (6), in rapid treatment decision-making, the participants' primary worries were not about themselves, but about who would take care of their families and how soon they could be cured and discharged (42). Thus, healthcare professionals who spend time with patients and provide comprehensive care during hospitalization, especially nurses, must be properly trained to provide consultation and empathetic interactions that will encourage participants to bravely disclose their inner emotions. These participants were also discovered to grapple with a great deal of confusion in their decision-making between mastectomy and breast-conserving surgery. Clearly, more accurate and clearer education and BC-related treatment information from healthcare professionals is greatly needed to guide appropriate decision-making.

Herein, participants frequently mentioned body-image disturbances. Consistent with previous studies (47), we found that among these participants, losing a breast was a terrible experience that made them think of themselves as fragmented. Likewise, among chemotherapy-related side effects, hair loss was considered among the most traumatizing and distressing experiences (8, 9). Many participants herein also felt uncomfortable in public because they were worried that others might recognize them as cancer patients from their asymmetrical breasts or hair loss (9, 48). Consequently, several participants were even distressed by dressing to go out and thus limited daily activities like exercise and shopping. It is unsurprising that body changes significantly impair body-image satisfaction, though this also appears to be a potential lifelong source of mental distress, including feeling less attractive and reduced self-esteem (8, 10, 15, 47). Therefore, healthcare professionals need to pay greater attention to body-image alterations of patients living with BC during both chemotherapy and surgical procedures; and should provide culturally appropriate psychosocial support or self-care strategy interventions regarding hair loss and altered body image.

Notably, we found that participants herein expressed significant concerns about fertility changes. These concerns may heighten guilt and lead to a self-identity crisis over the loss of gender roles. In China, the child is considered an important link between husband and wife, and a family without a new child is seen as incomplete (49). Interestingly, we summarized the different concerns over fertility of participants with different marital statuses influenced by traditional Chinese culture. Those who were married and childless had doubts about whether and when they could get pregnant after treatment. Those who invested a lot of energy and time into getting pregnant and were then diagnosed with BC were confronted with two simultaneous critical life events: one symbolizing the beginning of a new life, the other an existential threat to survival (6). Those who were married with a daughter felt great sorrow and self-blame over being unable to carry on the family line (i.e., failing to have a son) (50, 51) because the traditional Chinese concept of fertility—that a son is the status source of a married woman with her in-laws—persists in some areas of China (49). The three invisible shackles of “good wife, good mother, and filial daughter” within traditional Chinese culture mean that participants' emotional distress over role changes carries great significance. In China today, being married to a good wife remains a husband's most common psychological expectation, and being “a good wife” is also Chinese society's evaluation of a woman's competence (52, 53). In traditional Chinese culture, the master-slave nature of the husband-wife relationship dictates that the wife's duty and responsibility are meeting her husband's needs (53). Moreover, a filial daughter must fulfill a series of obligations to her family, making the necessary sacrifices to cultivate and perfect her virtue (52). In many people's opinion, being able to marry a good husband and have children are also regarded as a daughter's filial piety to her parents and parents-in-law (49, 50, 53). Furthermore, a loving mother is considered the standard of a good mother. Yet such unselfish, great love also fetters women's freedom and the choices they can make for themselves. When participants became sick, they had to set aside their employment and housework, shifting from a service provider to served. Such a changed gender role did not lead patients to realize “I am sick, I should rest and care for myself first”; instead, it strengthened their role consciousness of being “idle”, “a liability”, and “incompetent” (7, 54). Given their role in caring for patients, it is suitable for nurses to assess patient's concerns about sex and fertility, and to open a dialogue with them regarding these topics. This rapport can make it easier for patients to feel comfortable discussing their concerns about sensitive, intimate topics, and nurses can, in turn, provide patients with resources to help them better understand and address these anxieties.

The traditional Chinese virtues of bearing hardships and withstanding hard work can often be found in Chinese women (55, 56), so it is easy to understand the positive phenomenon of post-traumatic growth and cognitive reappraisal discovered herein. Examples of participants' post-traumatic growth include gains in self-esteem, self-confidence, and beliefs in their own potential, which might be carried over to future events (13, 14). The use of cognitive reappraisal led participants to face negative emotions instead of suppressing and escaping them. They became aware of a meaningfulness in maintaining an optimistic attitude. Moreover, they began to develop a renewed life with a new attitude, facing cancer and others' judgements bravely and confidently, and identifying themselves as achievers to enhance other's confidence in their ability to overcome cancer (13, 15). Looking back, many participants realized that “Life is very precious. We should take good care of ourselves” (2). They encouraged benefit finding and cognitive reappraisal recommending that other patients reassess their priorities and participate in activities that hold greater significance. Consistent with previous studies (21, 30), participants herein acknowledged that sufficient social support from their nation, family, friends, and peer patients were powerfully important buffers for coping with stress and helping reduce the harmful effects of negative events on their physical health and emotional wellbeing. Most participants expressed that the meticulous care from and constant company of their families gave them motivation and confidence to persist. This, in turn, strengthened their belief in cancer survival, for themselves and their families (2, 22). Thus, instead of considering this stressful event as an inescapable challenge, healthcare professionals may guide patients to assess it as a challenge that offers growth opportunity.

One of the impacts of cancer was that some participants were treated as “patients” and had great difficulties fulfilling their responsibilities maintaining their children's wellbeing and their families' stability (57). Consistent with Lu's report (43), several participants stated that they seldom disclosed their feelings to avoid placing emotional burdens on their family members. Others expressed that having no one understand them, and feeling so physically and mentally distressed and exhausted, was driving them to the point of insanity. Indeed, social support quality depends on an individual's ability to communicate their needs, while a lack of emotional disclosures may actually prevent others from detecting their support needs and reduce life satisfaction (21), further challenging early identification of their emotions. It is important to consider that participants may also feel misunderstood by others because they are bound by a traditional culture. Importantly, cultural proscriptions may stifle one's ability to seek support not only from friends and professionals, but from family members (21, 58). The Chinese perspective that there is a personal or intrinsically moral cancer cause discourages seeking medical advice and social support outside one's immediate circle because of social stigma (59). Chinese women are taught to be self-sufficient and to seek help only from within the family if they cannot cope with situations on their own (55). Seeking help outside the family indicates character weakness and is considered a shameful family disgrace (59, 60). Patients are both eager for targeted social support and afraid of being seen as weak and incompetent. This contradiction between inner needs and traditional culture leads to significant pain. Healthcare professionals should be aware of the conflicts that may arise in gender identity to fashion suitable programs to help and support patients as they transition between the roles of mother, wife, and daughter, to that of patient. Public health campaigns and psychoeducation about appropriate family support methods are also essential for patients' caregivers, so they can help patients transition from sickness to resilience.

By analyzing the interviews, EW, and Weibo text data, we found that although the three major themes and eight subthemes are presented across these sources, there were also some differences between them. The Weibo content tended to reflect a daily record of patients' lives and encouragement of themselves or others. In contrast, during the semi-structured interviews, participants tentatively asked questions in relation to issues about which they felt confused and were eager to address *via* direct communication (e.g., fertility, undergarments, surgical methods, choices regarding domestic or imported chemotherapeutic drugs). However, most noteworthy was the EW content. In both EW and the interviews, we encouraged patients to express the emotional experiences that bothered them most; however, EW seemed to promote patients' emotional disclosure and the EW contents were also richer than those of the interviews or Weibo.

Although we identified the theme of body-image disturbance and sense of stigma in the Weibo texts, we also found that the cause for this experience was often isolated to chemotherapy-related alopecia. In their EW texts, participants directly expressed a wider range of authentic feelings about their breast loss, such as, “I can't look at my breasts”, “I am no longer a real woman”, and “I dare not look in the mirror”. Furthermore, Weibo participants' expressions of worry about their role changes mainly centered on being unable to live normally because of physical dysfunction from surgery. In contrast, in EW texts, participants expressed guilt over role changes and focused on being unable to fulfill their responsibilities as wives, mothers, or daughters due to breast loss and long-term treatment. Participants' disbelief and an escape from reality after diagnosis could be found in all three data sources, but only in the interviews did we hear the voice of patients' regrets over delaying medical treatment due to a lack of disease awareness. Moreover, during those interviews, they encouraged women who feel abnormal in their bodies to have an early check-up. Importantly, our study identified the different concerns of participants relating to fertility being impacted by breast cancer treatment. These differed according to marital status and, given cultural expectations, may indicate a need to provide targeted emotional support to female Chinese patients with different marriage and childbearing concerns. As for the emotional trends in these data sources: Weibo texts tended to be more positive; it was obvious that participants preferred to remain calm during the interviews; and in the EW texts, negative emotions (i.e., self-complaints, doubts, helplessness, and fear) were very strong and clear.

That some cultural-specific emotional experiences identified *via* the three data sources herein reiterated those in previous studies with single-source data (5, 37, 61, 62) supports our hypothesis that there are few differences in self-report-based themes of emotional experiences by Chinese women, who tend to suppress emotions across methods of expression. Furthermore, considering that the participants herein were primarily experiencing an acute phase, when emotional distress and emotional fluctuation are most common, their reports likely reflect their strongest emotional experiences. That many emotional experiences reported herein were similar to those from previous studies that did not focus on disease phases (5, 10, 28, 61) emphasizes the long-term, persistent emotional distress common among patients with similar experiences and suggests that the emotional management and support they receive throughout the entire BC diagnosis and treatment process is insufficient or ineffective. Emotional communication modes preferred by Chinese women are non-verbal, indirect behaviors rather than direct verbalizations (59). Coincidentally, for patients with emotional distress and emotional needs, these data also show that EW and interviews are useful for both data collection and for patients to express their feelings and realize self-healing through emotional self-report and self-disclosure, further confirming the effectiveness of non-verbal, indirect behaviors. Thus, care providers should attend to levels of acculturation among patients living with BC to maintain clearer communication about treatment expectations and appropriate social support.

## 5. Strengths and limitations

This qualitative text analysis of data from three sources highlights the culture-based emotional experiences of female Chinese patients living with BC. We also analyzed the causes of these emotional experiences based on traditional Chinese culture and advanced corresponding care suggestions for healthcare professionals. Importantly, the theme and sub-themes summarized herein can nearly all be found within Weibo data alone, illustrating the validity and usefulness of this type of cost-effective Internet data for health research and providing a novel data source for future research analyzing patients' emotional experiences. Despite the novel and exciting nature of this study, we recognize several challenges with this analysis type. First, participants' sociodemographic characteristics were limited to the EW and interview participants, because personal information on Weibo network users is not publicly available. Thus, we were also unable to compare similarities or test differences in emotional experiences based on participant backgrounds. Second, most comments in Weibo are generally short and concise, and, although most of them are full of emotion, they are not conducive to theme coding. Third, some participants experience diagnosis, surgery, and chemotherapy within a short period, so their texts reflect their emotional experiences across phases. Due to the difficulty analyzing their emotional experiences during the three different phases separately, we included all the patients in all three phases as a whole and the results thus reflect patients' emotions overall.

## 6. Conclusions

The emotional distress suffered by patients with BC can be varied, long-term, and extremely challenging. However, when women engaged actively in benefit finding and cognitive reappraisal with strong social support, patients were able to find ways to adapt and reported post-traumatic growth. Strong social support was an important facilitator in this growth. Therefore, healthcare professionals need to understand and respect these unique experiences and must provide these patients with psychosocial counseling and timely, tailored, culturally appropriate emotional support. This is a potential target for nursing interventions. Moreover, female Chinese patients with BC are generally restrained in their emotional expressions and generally reluctant to reveal their feelings. It is therefore valuable for healthcare professionals to increase cultural sensitivity when providing services to female patients with BC who are from diverse cultural backgrounds. Furthermore, it is essential to screen for distress symptoms in patients at all BC diagnostic and treatment phases as part of the standard nursing care routine. Additionally, these data support the provision of different channels to encourage female patients with BC to express their emotions, and they show that exploring validated, reliable measures of emotion expression, body image, sexuality, and fertility is warranted for healthcare professionals.

## Data availability statement

The original contributions presented in the study are included in the article/Supplementary material, further inquiries can be directed to the corresponding author.

## Ethics statement

The research has been conducted in accordance with the Declaration of Helsinki and following ethical principles and guidelines. Ethics approval for the study was granted by the Medical Ethics Committee of Nanfang Hospital of Southern Medical University (NFEC-2021-124) and a standardized informed consent form had been established (V1.0/2021-4-14). Eligible participants were informed about the study and provided written informed consent for participation prior to the study.

## Author contributions

CL, CU, WZ, CZhe, JLi, and YW: conception, design, and revising the article critically for intellectual content. CL, WZ, CZhe, JLi, CZho, BJ, LS, WL, LX, YM, HZ, YL, JLa, and JF: acquisition of data. CL, WZ, CZhe, and JLi: analysis, interpretation of data, and drafting the article. CL, CU, and YW: writing-review and editing. All authors contributed to the article and approved the submitted version.
