# Supplementary material for: Listening to voices from multiple sources: A qualitative text analysis of the emotional experiences of women living with breast cancer in China
Source: Front Public Health. 2023 Feb 3;11:1114139. doi: 10.3389/fpubh.2023.1114139 (PMC9935709; doi:10.3389/fpubh.2023.1114139)
Supplement: Supplementary file 1 [file Table_1.pdf]

Supplementary File 1 The consolidated criteria for reporting qualitative studies (COREQ) of this study

No	Item	Guide questions/description	Location where item is reported
**Domain 1: Research team and reflexivity**			
Personal Characteristics			
1	Interviewer/facilitator	Which author/s conducted the interview or focus group?	Page 5 of the Manuscript identify the facilitators.
2	Credentials	What were the researcher's credentials? E.g., PhD, MD	Page 5 of the Manuscript identify the researcher's credentials
3	Occupation	What was their occupation at the time of the study?	Page 5 of the Manuscript identify the researcher's occupation.
4	Gender	Was the researcher male or female?	Page 5 of the Manuscript identify the researcher's gender.
5	Experience and training Relationship with participants	What experience or training did the researcher have?	Page 5 of the Manuscript identify the experience or training the researcher have.
6	Relationship established	Was a relationship established prior to study commencement?	Page 5 describes the researcher's occupation and relationship established prior to study commencement for they are part of the staff of the research department.
7	Participant knowledge of the interviewer	What did the participants know about the researcher? e.g., personal goals, reasons for doing the research	Page 5 and Table 3 of the Manuscript specify the participant knowledge of the interviewer and expressive writing.
8	Interviewer characteristics	What characteristics were reported about the interviewer/facilitator? e.g., Bias, assumptions, reasons, and interests in the research topic	Page 5 of the Manuscript specify the interviewer and expressive writing characteristics.
**Domain 2: study design**			
Theoretical framework			
9	Methodological orientation and Theory	What methodological orientation was stated to underpin the study? e.g., grounded theory, discourse analysis, ethnography, phenomenology, content analysis	We choose the phenomenology to underpin the study. See details in the references 34-36 on Page 5 or Page 20.

Supplementary File 1 (continued)

No	Item	Guide questions/description	Location where item is reported
Participant selection			
10	Sampling	How were participants selected? e.g., purposive, convenience, consecutive, snowball	See details in page 5 about the sampling method.
11	Method of approach	How were participants approached? e.g., face-to-face, telephone, mail, email	See details in pages 5 and 6 about the participants and setting and data collection.
12	Sample size	How many participants were in the study?	Page 7 provides an explicit statement about the sample size.
13	Non-participation How many people refused to participate or dropped out? Reasons?	How many people refused to participate or dropped out? Reasons?	N/A. Because no people refused to participate or dropped out in our study.
Setting			
14	Setting of data collection	Where was the data collected? e.g., home, clinic, workplace	Pages 5 provide an explicit statement about the setting of data collection.
15	Presence of non-participants	Was anyone else present besides the participants and researchers?	Pages 5 specify the setting of data collection. Except that the interview was conducted alone in an undisturbed room, the expressive writing was conducted in patients' unit where there was someone else present besides the participants and researchers.
16	Description of sample	What are the important characteristics of the sample? e.g., demographic data, date	Table 2 on page 24 provides an explicit demographic characteristics of participants in expressive writing and interview, and pages 16 states the reason why we didn't show the demographic data of the Weibo participants.
17	Interview guide	Were questions, prompts, guides provided by the authors? Was it pilot tested?	Table 3 specify the expressive writing and interview guide.
18	Repeat interviews	Were repeat interviews carried out? If yes, how many?	N/A. No repeat interviews were carried out.
19	Audio/visual recording	Did the research use audio or visual recording to collect the data?	Page 6 describes that we collected interview data in the form of audio recording.
20	Field notes	Were field notes made during and/or after the interview or focus group?	Page 6 describes the field notes and memos we made to support the connection between the data and findings after the interview.

Supplementary File 1 (continued)

No	Item	Guide questions/description	Location where item is reported
21	Duration	What was the duration of the interviews or focus group?	Page 5 depicts the duration of the interviews.
22	Data saturation	Was data saturation discussed?	Pages 5 discuss the data saturation.
23	Transcripts returned	Were transcripts returned to participants for comment and/or correction?	N/A. In the interview, we confirmed the contents stated by the participants, and researchers can repeatedly confirm the information obtained from Weibo and expressive writing.
**Domain 3: analysis and findings**			
Data analysis			
24	Number of data coders	How many data coders coded the data?	Page 6 depicts the number of data coders.
25	Description of the coding tree	Did authors provide a description of the coding tree?	N/A. Page 6 depicts the coding process.
26	Derivation of themes	Were themes identified in advance or derived from the data?	Pages 6 and 7 depicts the derivation of themes.
27	Software	What software, if applicable, was used to manage the data?	Page 6 specifies the software we use.
28	Participant checking	Did participants provide feedback on the findings?	Participants don't provide feedback on the findings.
Reporting			
29	Quotations presented	Were participant quotations presented to illustrate the themes / findings? Was each quotation identified? e.g., participant number	Page 7-12 and Supplementary File 2 provide an explicit statement about the participant quotations to illustrate the themes.
30	Data and findings consistent	Was there consistency between the data presented and the findings?	Page 7-16 describes the consistency between the data presented and the findings.
31	Clarity of major themes	Were major themes clearly presented in the findings?	Page 7-12 and Supplementary File 2 provide a clear statement about the major themes.
32	Clarity of minor themes	Is there a description of diverse cases or discussion of minor themes?	Page 7-12 provides an explicit description of diverse cases of minor themes.
