# Supplementary material for: Listening to voices from multiple sources: A qualitative text analysis of the emotional experiences of women living with breast cancer in China
Source: Front Public Health. 2023 Feb 3;11:1114139. doi: 10.3389/fpubh.2023.1114139 (PMC9935709; doi:10.3389/fpubh.2023.1114139)
Supplement: Supplementary file 2 [file Table_2.pdf]

Supplementary File 2 Themes, sub-themes, and representative participant quotes along with the number and proportion of participants who mentioned each theme and sub-theme

**Themes (n,%)**	**Sub-themes**	**Examples of quotes**
Conflicting emotions after diagnosis (804, 32.7%)	Concerns about disclosure cancer diagnosis (158, 6.4%)	"I felt very uncomfortable after the operation, while when my parents called me, I pretended that it was just a minor operation, for I knew if my mother knew, she would not be able to bear the pain. Thus, I'm choking back myself, actually, I'm suffering physically and mentally." [PD11]
		"Many people around me didn't know that I am ill, and I don't want to tell them because it doesn't make any sense. Everyone is in pain. I want to bring more happiness to them. [PO30]
		"Many relatives didn't know I was sick except the closest ones. I really didn't want to disclose my illness, and burden anyone who cares about me. Moreover, I hate being pitied by others!" [PC34]
		"I don't want to disclose my diagnosis. I am not a complete woman anymore and I'm afraid that they will laugh at me. Therefore, I have to conceal my illness." [PW14]
		"I always think that getting sick is my own business, I don't want to be given unnecessary sympathy and be gossiped by others." [PI17]
	Disbelief and an escape from reality (187, 7.6%)	"I experienced a thunderbolt-like announcement, and realized various moods mixed with fear, helplessness, grievance, worry. I will be helpless to cry, anxious to lose sleep." [PD6]
		"The doctor said to me, 'The result is as malignant as we expected.' At that moment, the dark curtain that had been hanging over the sky suddenly seemed to fall, wrapping my body, and tightening my throat." [PD41]
		"After reading the diary written two days ago, I felt more stressed. In short, I didn't want to see any information about BC, and I don't want to face my illness." [PC12]
		"I was in a very bad mood when I knew the diagnosis, I felt like the sky was going to fall. How could I survive in the future? I might die. I really didn't want to live, so miserable! It' was really unfair to be a woman." [PC26]

Supplementary File 2 (continued)

**Themes**	**Sub-themes**	**Examples of quotes**
		Sometimes, I was lost in thought, I haven't done anything against my conscience, but why did this disaster fall on me? Why did I get the disease?" [PI10]
		"Why did I have the cancer? I couldn't believe it. I cried. I was scared. I never thought that cancer would happen to me! I'm so young, why?" [PW7]
		Although I already saw the diagnosis report, I didn't believe the examination, it must be misdiagnosed, how can I be breast cancer? Thus, I transferred to another hospital and had the frozen section examined again. Unexpectedly, the diagnosis was BC as last time, but I still didn't want to believe it." [PW479]
	Distress over rapid treatment decision-making (459, 18.7%)	"The doctor asked if I hesitated to have an operation, I answered him 'yes' firmly, because I thought if it was benign, then there would be no need to have the mastectomy. If the breast was resected, it would disappear and couldn't be sewn up forever (helpless, with tears shining in my eyes)." [PD10]
		"During the operation, the mass of my breast was diagnosed as cancer. The doctor advised me to resect it, but I didn't want to do so, I just kept crying in the operating room." [PO15]
		"The whole breast has to be removed, which makes me feel sorrowful and grieved ⋯ However, without life, everything is meaningless. Therefore, if losing a breast can bring back my life, I can try to accept this fact." [PC3]
		"I'm still hesitant and worried, because I think that the recurrence risk of different surgical methods varied." [PW9]
		"I was a little anxious at the time when I was diagnosed, and I couldn't sleep at night. What I concerned was not the cancer itself, but which hospital I should go for treatment." [PI8]
		"Because I have been hesitating to choose the operation method, I really can't accept the mastectomy. If the doctor tells me there was a chance to preserve the breast. I will choose breast-conserving surgery definitely." [PI9]

Supplementary File 2 (continued)

Themes	Sub-themes	Examples of quotes
Long-term suffering and treatment concerns (953, 38.8%)	Body image disturbance and sense of stigma (277, 11.3%)	"I dare not take off my clothes and look at myself in the mirror, and I dare not face my fragmentary body clearly until now." [PO14]
		Clothes that are too thin or tight, with buttons on the chest... all can't be worn because my ugly asymmetrical breast and "crippled" arm. Consequently, my good mood was destroyed by the unsatisfactory process of dressing, and I finally have no desire to go out. [PO33]
		"I was afraid of being regarded as an infectious person. My mother-in-law gave me a bowl to eat with them separately. Although it seemed to be for the sake of the health of the whole family, I couldn't help crying, and my heart was full of panic and loneliness." [PC27]
		"Whenever I take a shower and see my broken breasts, my mood suddenly becomes very bad. Although my husband doesn't care about my body image, I always feel that I am an imperfect woman." [PC40]
		"When I see my bald head, I feel ugly, I hate this appearance. I don't know how long it will last; I don't want to go out because I think others are looking at me in strange eyes." [PW2]
		"I haven't written for a long time, my hands are out of control, my handwriting is really ugly, just like me, an ugly monster." [PI2]
	Guilt and powerlessness over gender role loss and conflict (396, 16.1%)	"During hospitalization, I didn't care what kind of fear I was about to face. I even thought that death might be a relief without troubles or pains, I just fell asleep forever. But my children, my elderly mother, all my family and relatives were my biggest worry and couldn't give up." [PD25]
		"Will I die? I don't know who will take care of my young children if I die. How can they live without mother's company? I worry they will be bullied by others. So pitiful!" [PD37]
		"When I come back home, I still have to do housework as before, because there is no one mopping the floor or washing the dishes. Moreover, my husband is very lazy. All these are overwhelming me. I even think breathing is very painful." [PO9]

Supplementary File 2 (continued)

**Themes**	**Sub-themes**	**Examples of quotes**
		"I feel sorry for my kind husband. It would be great if we hadn't met each other, he could find a healthy and beautiful woman. If one day I die, I hope he can find a healthy person to be his wife. I am destined to owe him." [PO29]
		"I also hate myself now, as if I were a grumbling woman, just complaining my helplessness every day. My life circle is really too small that I am sure that I will be alienated from society, and even my husband if I always in such a situation." [PO34]
		"Seeing my husband busy for our livelihood, while I can't help anything. Sometimes I ask him, am I a burden to you?" [PC19]
		"I really hate myself for being useless, encumbering my son to school, encumbering my husband to make money, encumbering my family, encumbering my parents-in-law... I'm afraid I can't live up to their expectations." [PC20]
		"One month after the operation, the doctor still doesn't recommend upper limb abduction. I'm worried that if I don't exercise, it will become more difficult for my arm to return to normal? Now I'm a little hunched!" [PW4557]
	Anxiety about sexuality and fertility changes (280, 11.4%)	"I'm 36 years old and haven't had a baby yet. Now I've had a mastectomy, not to mention how ugly my image is. What worries me most is my future sex life. Additionally, how should I adapt to the reality that I can't breastfeed?" [PD49]
		"Since the operation and chemotherapy, I have been indifferent to sex. I usually have sex two or three times a month, but now one is redundant." [PC20]
		"I lost my sex life after I got the cancer. I sleep in a separate room with my husband. Maybe he thinks I'm not perfect. I'm a monster with no breasts··· Monster···" [PC47]
		"It's nothing else that makes me burst into tears suddenly, but I'm concerned about having a baby. I'm not sure whether I can have a baby. The doctor said it would be a few years later, but I don't know exactly how long later." [PW1762]

Supplementary File 2 (continued)

Themes	Sub-themes	Examples of quotes
		"Seeing my sister's happiness after pregnancy, I really admire her. I guess I will never have the lucky chance in my life." [PW2394]
		"Watching others play with children in the street makes me instantly envious. But now. . . Will anyone like me in the future? Will I possess my own happiness?" [PW3790]
		"I just got married and haven't given birth when I was diagnosed, but now my husband is in infertility, so I want to ask if anyone knows about the fertility problem after breast cancer is cured." [PW5387]
Benefit finding and cognitive reappraisal (748, 30.4%)	Post-traumatic growth (582, 23.7%)	"This illness is an unforgettable experience. Everyone is vulnerable, only when she bravely accepts all the frustrations and pains brought by fate can she gain the new growth" [PD12]
		"I try to receive all changes. Now I am no longer anxious and helpless. I am ready to welcome the unpredictable future calmly. Physically or psychologically, I will be my own master." [PC5]
		"All this pain has survived! It's sunny now! A good life gives us the impetus to move forward! Painful memories wash away our chaotic souls!" [PW4121]
		"What impressed me most is the communication between patients, which has become my motivation to overcome everything! Everyone has different experiences, worries, and difficulties, but the same goal is that we are survive for our families. In order not to burden our families, we cheer up together and form a slogan of "Come on! Victory is ours." [PC31]
		"The suffering we are experiencing is only temporary, and we are moving towards the end of victory. Compared with those who are always suffering, I am lucky enough." [PC25]

Supplementary File 2 (continued)

**Themes**	**Sub-themes**	**Examples of quotes**
	Perception of social support (166, 6.8%)	"When I was sick, my husband was my strongest support, his daily comfort and encouragement made met greatly moved. Before I came to the hospital, I told him about my concerns towards future life, but he always tried his best to encourage me." [PD26]
		"Because of the cancer and painful scar, I had been in a bad mood when I went home after the operation. I didn't want to eat and often lost my temper. Fortunately, my husband didn't mind my losing my temper with him., he always tried to make me happy, cook meals I like, and encourage me, which made me embarrassed to get angry, but grateful." [PO11]
		"Through the project of 'National Screening for Cervical Cancer and Breast Cancer', I was diagnosed with BC. Thus, I would like to thank our country for her care and concern for all women, the hospital for its advanced medical technology, and all the medical staff." [PO12]
		"Seeing that my appetite is getting better, my family cook different delicious food every day, such as stewed chicken soup and abalone. Besides being moved, I occasionally feel guilty… It's really my blessing and luck to have such a caring family." [PC16]
		"Because of the chemotherapy, the whole family pays more attention to my nutrition than before, and every meal is carefully prepared and matched, so happy! I want to recover soon, which I think is the best reward for them!" [PW733]

* PD1-50: Newly diagnosed phase participants; PO1-50: Post-operative phase participants; PC1-50: Chemotherapy phase participants; PW1-5,678:Weibo participants; PI1-17: Semi-structured interview participants.
