# Supplementary material for: Listening to voices from multiple sources: A qualitative text analysis of the emotional experiences of women living with breast cancer in China
Source: Front Public Health. 2023 Feb 3;11:1114139. doi: 10.3389/fpubh.2023.1114139 (PMC9935709; doi:10.3389/fpubh.2023.1114139)
Supplement: Supplementary file 3 [file Table_3.pdf]

Supplementary File 3 The proportion of themes and sub-themes mentioned in each source

**Themes (n,%)**	Semi-structured interview (N_1=17)	Expressive writing (N_2=150)	Weibo (N_3=2290)	**Sub-themes (n,%)**	Semi-structured interview (N_1=17)	Expressive writing (N_2=150)	Weibo (N_3=2290)
Conflicting emotions after diagnosis	(15, 88.2%)	(117, 78.0%)	(672, 24.2%)	Concerns about disclosure cancer diagnosis	(5, 29.4%)	(105, 70.0%)	(48, 2.1%)
				Disbelief and an escape from reality	(15, 88.2%)	(129, 86.0%)	(43, 1.9%)
				Distress over rapid treatment decision-making	(12,70.6%)	(132, 88.0%)	(315, 13.8%)
Long-term suffering and treatment concerns	(12, 70.6%)	(121, 80.7%)	(820, 35.8%)	Body image disturbance and sense of stigma	(7, 41.2%)	(122, 81.3%)	(148, 6.5%)
				Guilt and powerlessness over female role loss and conflict	(13, 76.5%)	(135, 90.0%)	(248, 10.8%)
				Anxiety about sexuality and fertility changes	(12, 70.6%)	(108, 72.0%)	(160, 7.0%)
Benefit finding and cognitive reappraisal	(9, 52.9%)	(78, 52.0%)	(661, 28.9%)	Post-traumatic growth	(13, 76.5%)	(122, 81.3%)	(447, 19.5%)
				Perception of social support	(6, 35.3%)	(46, 30.7%)	(114, 5.0%)

* N_1: the number of participants from semi-structured interview; N_2: the number of participants from expressive writing; N_3: the number of participants from Weibo.
